# An approximation of populations on a habitat with large carrying capacity

**DOI:** 10.1007/s00285-024-02069-w

**Published:** 2024-03-18

**Authors:** Naor Bauman, Pavel Chigansky, Fima Klebaner

**Affiliations:** 1 Department of Statistics, The Hebrew University of Jerusalem, Mount Scopus Campus, 9190501 Jerusalem, Israel; 2 School of Mathematics, Monash University, Clayton, VIC 3058 Australia

**Keywords:** Population dynamics, Branching processes, Limit theorems, Approximation, 92D25, 60J80

## Abstract

We consider stochastic dynamics of a population which starts from a small colony on a habitat with large but limited carrying capacity. A common heuristics suggests that such population grows initially as a Galton–Watson branching process and then its size follows an almost deterministic path until reaching its maximum, sustainable by the habitat. In this paper we put forward an alternative and, in fact, more accurate approximation which suggests that the population size behaves as a special nonlinear transformation of the Galton–Watson process from the very beginning.

## Introduction

### The model

A large population often starts from a few individuals who colonize a new habitat. Initially, in abundance of resources and lack of competition it grows rapidly until reaching the carrying capacity. Then the population fluctuates around the carrying capacity for a very long period of time, until, by chance, it eventually dies out, see, e.g., Haccou et al. (2007); Hamza et al. (2016).

This cycle is captured by a stochastic model of density dependent branching process $Z=(Z_n, n\in {\mathbb {Z}}_+)$ generated by the recursion

1 $$\begin{aligned} Z_n = \sum _{j=1}^{Z_{n-1}} \xi _{n,j}, \quad n\in {\mathbb {N}}, \end{aligned}$$

started at an initial colony size $Z_0$. The random variables $\xi _{n,j}$ take integer values and, for each $n\in {\mathbb {N}}$, are conditionally i.i.d. given all previous generations

$$\begin{aligned} {\mathcal {F}}_{n-1} =\sigma \{\xi _{m,j}: m<n, j\in {\mathbb {N}}\}. \end{aligned}$$

The object of our study is the *density* process of the population ${{\overline{Z}}}_{n}:= Z_{n}/K$ relative to the carrying capacity parameter $K>0$. The common distribution of the random variables $\xi _{n,j}$ is assumed to depend on the density ${{\overline{Z}}}_{n-1}$:

2 $$\begin{aligned} {\textsf{P}}(\xi _{n,1}=\ell |{\mathcal {F}}_{n-1}) = p_\ell ({{\overline{Z}}}_{n-1}), \quad \ell \in {\mathbb {Z}}_+, \end{aligned}$$

and is determined by the functions $p_\ell :{\mathbb {R}}_+\mapsto [0,1]$.

Both processes *Z* and ${{\overline{Z}}}$ are indexed by *K*, but this dependence is suppressed in the notation. The mean and the variance of offspring distribution when the density process has value *x* are denoted by

3 $$\begin{aligned} m(x) = \sum _{k=0}^\infty k p_k(x)\quad \text {and}\quad \sigma ^2(x) = \sum _{k=0}^\infty (k-m(x))^2 p_k(x), \quad x\in {\mathbb {R}}_+, \end{aligned}$$

assumed to exist. Consequently,

$$\begin{aligned} {\textsf{E}}(\xi _{n,1}|{\mathcal {F}}_{n-1}) = m({{\overline{Z}}}_{n-1}) \quad \text {and}\quad \textsf{Var}(\xi _{n,1}|{\mathcal {F}}_{n-1})=\sigma ^2({{\overline{Z}}}_{n-1}). \end{aligned}$$

If the offspring mean function satisfies

4 $$\begin{aligned} m(x) {\left\{ \begin{array}{ll}> 1, &{} x<1\\ =1, &{} x=1\\ <1, &{} x>1 \end{array}\right. } \end{aligned}$$

the process *Z* has a supercritical reproduction below the capacity *K*, critical reproduction at *K* and a subcritical reproduction above *K*. Thus a typical trajectory of *Z* grows rapidly until it reaches the vicinity of *K*. It then stays there fluctuating for a very long period of time and gets extinct eventually if $p_0(x)>0$ for all $x\in {\mathbb {R}}_+$. Thus the lifespan of such population roughly divides between the emergence stage, at which the population establishes itself, the quasi-stationary stage around the carrying capacity and the decline stage which ends up with inevitable extinction.

#### Remark 1

While (4) is typical for populations with quasi stable equilibrium at the capacity, it is not needed in the proofs and will not be assumed in what follows.

### Large initial colony

A more quantitative picture can be obtained by considering the dynamics for the density process derived from (1) by setting $f(x):=xm(x)$, dividing by *K* and rearranging:

5 $$\begin{aligned} {{\overline{Z}}}_n = f({{\overline{Z}}}_{n-1}) + \frac{1}{K} \sum _{j=1}^{Z_{n-1}} (\xi _{n,j}-m({{\overline{Z}}}_{n-1})). \end{aligned}$$

The second term on the right has zero mean and conditional variance

$$\begin{aligned} \textsf{Var}\left( \frac{1}{K} \sum _{j=1}^{Z_{n-1}} (\xi _{n,j}-m({{\overline{Z}}}_{n-1}))\Big |{\mathcal {F}}_{n-1}\right) =K^{-1}{{\overline{Z}}}_{n-1} \sigma ^2({{\overline{Z}}}_{n-1}). \end{aligned}$$

Consequently (5) can be viewed as a deterministic dynamical system perturbed by small noise of order^1^$O_{\textsf{P}}(K^{-1/2})$. If the initial colony size is relatively large, i.e., proportional to the carrying capacity:

$$\begin{aligned} {{\overline{Z}}}_0=Z_0/K \xrightarrow [K\rightarrow \infty ]{} x_0>0, \end{aligned}$$

then ${{\overline{Z}}}_n \xrightarrow [K\rightarrow \infty ]{{\textsf{P}}} x_n$ where $x_n$ follows the unperturbed deterministic dynamics

6 $$\begin{aligned} x_n = f(x_{n-1}), \quad n\in {\mathbb {N}}, \end{aligned}$$

started at $x_0$. If (4) is assumed, $x=1$ is the stable fixed point of *f* and if, in addition, *f* is an increasing function, then the sequence $x_n$ increases to 1 with *n* when $x_0<1$. This limit also implies that the probability of early extinction tends to zero as $K\rightarrow \infty$.

Moreover, the stochastic fluctuations about the deterministic limit converge to a Gaussian process $V=(V_n, n\in {\mathbb {Z}}_+)$ in distribution:

$$\begin{aligned} \sqrt{K} ({{\overline{Z}}}_n-x_n)\xrightarrow [K\rightarrow \infty ]{d}V_n \end{aligned}$$

where $V_n$ satisfies the recursion, Klebaner and Nerman (1994),

$$\begin{aligned} V_n = f'(x_{n-1}) V_{n-1} + \sqrt{x_{n-1} \sigma ^2(x_{n-1})}W_n,\quad n\in {\mathbb {N}}, \end{aligned}$$

with *N*(0, 1) i.i.d. random variables $W_n$’s.

Roughly speaking, this implies that when *K* is large, $Z_n$ grows towards the capacity *K* along the deterministic path $K x_n$ and its fluctuations are of order $O_{\textsf{P}}(K^{1/2})$:

7 $$\begin{aligned} Z_n = x_n K + V_n K^{1/2} + o_{\textsf{P}}(K^{1/2}), \quad n\in {\mathbb {N}}. \end{aligned}$$

If $p_0(x)>0$ for all $x\in {\mathbb {R}}_+$ and (4) is imposed, zero is an absorbing state and hence the population gets extinct eventually. Large deviations analysis, see for example Klebaner and Zeitouni (1994); Klebaner et al. (1998), and Jung (2013); Högnäs (2019), shows that the mean of the time to extinction $\tau _e =\inf \{n\ge 0: Z_n=0\}$ grows exponentially with *K*. In this paper we are concerned with the establishment stage of the population, which occurs well before the ultimate extinction, on the time scale of $\log K$.

### Small initial colony

When $Z_0$ is a fixed integer, say $Z_0=1$, then $Z_0/K\rightarrow x_0=0$ and, since $f(0)=0$, the solution to (6) is $x_n=0$ for all $n\in {\mathbb {N}}$. In this case the approximation (7) ceases to provide useful information. An alternative way to describe the stochastic dynamics in this setting was suggested recently in Barbour et al. (2016); Chigansky et al. (2018, 2019). It is based on the long known heuristics (Kendall 1956; Whittle 1955; Metz 1978), according to which such a population behaves initially as the Galton–Watson branching process and, if it manages to avoid extinction at this early stage, it continues to grow towards the carrying capacity following an almost deterministic curve.

This heuristics is made precise in Chigansky et al. (2019) as follows. We couple *Z* to a supercritical Galton–Watson branching process $Y=(Y_n, n\in {\mathbb {Z}}_+)$ started at $Y_0=Z_0=1$,

8 $$\begin{aligned} Y_n = \sum _{j=1}^{Y_{n-1}} \eta _{n,j} \end{aligned}$$

with the offspring distribution identical to that of *Z* at zero density size

$$\begin{aligned} {\textsf{P}}(\eta _{1,1}=\ell ) = p_\ell (0), \quad \ell \in {\mathbb {Z}}_+. \end{aligned}$$

This coupling is defined under assumption (a1.) below in Sect. 3.2.

Denote by $\rho :=m(0)>1$, define^2^$n_c:= n_c(K)=[\log _\rho K^c]$ for some $c\in (\frac{1}{2},1)$ and let ${{\overline{Y}}}_n:=Y_n/K$ be the density of *Y*. Then ${{\overline{Z}}}_n=Z_n/K$ is approximated in Chigansky et al. (2019) by

$$\begin{aligned} {\left\{ \begin{array}{ll} {{\overline{Y}}}_n,&{} n\le n_c, \\ f^{n-n_c}({{\overline{Y}}}_{n_c}), &{} n>n_c, \end{array}\right. } \end{aligned}$$

where $f^k$ stands for the *k*-th iterate of *f*. As is well known (Athreya and Ney 1972)

$$\begin{aligned} \rho ^{-n} Y_n \xrightarrow [n\rightarrow \infty ]{{\textsf{P}}-\text {a.s.}} W, \end{aligned}$$

where *W* is an a.s. finite random variable. Moreover, under certain technical conditions on *f*, the limit

9 $$\begin{aligned} H(x):=\lim _{n\rightarrow \infty }f^n(x/\rho ^n), \quad x\in {\mathbb {R}}_+ \end{aligned}$$

can be shown to exist and define a continuous function.

#### Theorem 1

(Chigansky et al. 2019) Let $n_1:=n_1(K)=[\log _\rho K]$ then

10 $$\begin{aligned} {{\overline{Z}}}_{n_1} - H\Big (W\rho ^{-\{\log _\rho K\}}\Big ) \xrightarrow [K\rightarrow \infty ]{{\textsf{P}}}0. \end{aligned}$$

In particular, this result implies that when *K* is a large integer power of $\rho$ the distribution of ${{\overline{Z}}}_{n_1}$ is close to that of *H*(*W*). Moreover,

$$\begin{aligned} {{\overline{Z}}}_{n_1+n}\xrightarrow [K\rightarrow \infty ]{{\textsf{P}}} x_n, \quad n\in {\mathbb {N}}, \end{aligned}$$

where $x_n$ solves (6) started from the *random* initial condition *H*(*W*). This approximation also captures the early extinction event since $H(0)=0$ and ${\textsf{P}}(W=0)={\textsf{P}}(\lim _n Y_n=0)$, the extinction probability of the Galton–Watson process *Y*.

### This paper’s contribution

In this paper we address the question of the rate of convergence in (10). Note that if the probabilities in (2) are constant with respect to *x* then $f(x)=\rho x$, consequently $H(x)=x$, and the processes *Z* and *Y* coincide. In this case

11 $$\begin{aligned} \sqrt{K} ({{\overline{Y}}}_{n_1} -W\rho ^{-\{\log _\rho K\}}) = \rho ^{-\frac{1}{2}\{\log _\rho K\}} \sqrt{\rho ^{n_1}} (\rho ^{-n_1}Y_{n_1} -W) = O_{\textsf{P}}(1) \end{aligned}$$

where the order of convergence is implied by the CLT for the Galton–Watson process (Heyde 1970) by which $\sqrt{\rho ^{n}} (\rho ^{-n}Y_{n} -W)$ converges in distribution to a mixed normal law as $n\rightarrow \infty$. Thus it can be expected that at best the sequence in (10) is of order $O_{{\textsf{P}}}(K^{-1/2})$ as $K\rightarrow \infty$. However, the best rate of convergence in the approximation in Theorem 1 described above, is achieved with $c=\frac{5}{8}$ and it is only $O_{\textsf{P}}(K^{-1/8}\log K)$. This can be seen from a close examination of the proof in Chigansky et al. (2019).

The goal of this paper is to put forward a different approximation with much faster rate of convergence of order $O_{\textsf{P}}(K^{-1/2}\log K)$. This is still slower than the rate achievable in the density independent case, but only by a logarithmic factor. The new proof highlights a better understanding of population dynamics at the emergence stage, which shows that, in fact, a sharper approximation is given by the Galton–Watson process transformed by a nonlinear function *H* arising in deterministic dynamics (9).

It is not clear at the moment whether the $\log K$ factor is avoidable and whether a central limit type theorem holds. These questions are left for further research.

## The main result

We will make the following assumptions.

1. The offspring distribution $F_x(t) = \sum _{\ell \le t} p_\ell (x)$ is stochastically decreasing with respect to the population density: for any $y\ge x$,
  $$\begin{aligned} F_y(t)\ge F_x(t), \quad \forall t\in {\mathbb {R}}_+. \end{aligned}$$
2. The second moment of the offspring distribution, cf. (3),
  $$\begin{aligned} m_2(x) = \sigma ^2(x)+m(x)^2 \end{aligned}$$
  is *L*-Lipschitz for some $L>0$.
3. The function $f(x)=xm(x)$ has two continuous bounded derivatives and^3^
  $$\begin{aligned} \Vert f'\Vert _\infty = f'(0)=\rho . \end{aligned}$$

### Remark 2

Assumption (a1.) means that the reproduction drops with population density. In particular, it implies that $x\mapsto m(x)$ is a decreasing function and hence,

$$\begin{aligned} f'(x)=m(x)+xm'(x) \le m(x)\le \rho ,\quad \forall x\in {\mathbb {R}}_+, \end{aligned}$$

which is only slightly weaker than (a3.). The assumption (a2.) is technical.

### Remark 3

The distribution of the process ${{\overline{Z}}}$ does not depend on the values of $\{p_\ell (0), \ \ell \in {\mathbb {Z}}_+\}$ for any *K*, while the distribution of *W* and, therefore, of *H*(*W*) does. This is not a contradiction since our assumptions imply continuity of $x\mapsto p_\ell (x)$ at $x=0$ for all $\ell \in {\mathbb {Z}}_+$. Indeed, $m(x) = \int _0^\infty (1-F_x(t))dt$ and therefore

$$\begin{aligned} \int _0^\infty (F_x(t)-F_0(t))dt = m(0)-m(x)\xrightarrow [x\rightarrow 0]{}0 \end{aligned}$$

where the convergence holds since *m*(*x*) is differentiable and a fortiori continuous at $x=0$. By the stochastic order assumption (a1.), $F_x(t)-F_0(t)\ge 0$ for any $t\ge 0$. Since both $F_x$ and $F_0$ are discrete with jumps at integers, for any $s\ge 0$,

$$\begin{aligned} F_x(s)-F_0(s) = \int _{[s]}^{[s]+1} (F_x(t)-F_0(t))dt\le \int _0^\infty (F_x(t)-F_0(t))dt \xrightarrow [x\rightarrow 0]{} 0. \end{aligned}$$

This in turn implies that $p_\ell (x)\rightarrow p_\ell (0)$ as $x\rightarrow 0$ for all $\ell$.

### Theorem 2

Under assumptions (a1.)–(a3.)

$$\begin{aligned} {{\overline{Z}}}_{n_1} - H\Big (W\rho ^{-\{\log _\rho K\}}\Big ) = O_{\textsf{P}}\big (K^{-1/2}\log K\big ), \quad \text {as }\,\ K\rightarrow \infty . \end{aligned}$$

### Example 1

The binary splitting model from Chigansky et al. (2018) satisfies the above assumptions. Another example is Geometric offspring distribution

$$\begin{aligned} p_\ell (x) = q(x)^\ell (1-q(x)), \quad \ell \in {\mathbb {Z}}_+ \end{aligned}$$

where $q:{\mathbb {R}}_+\mapsto [0,1]$ is a decreasing function. This distribution satisfies the stochastic order condition (a1.). The normalization $m(0)=\rho$ and $m(1)=1$ implies that $q(0)=\rho /(1+\rho )$ and $q(1)=1/2$. A direct calculation shows that, e.g.,

$$\begin{aligned} q(x) = \frac{\rho }{1+\rho }\exp \left( -x \log \frac{2\rho }{1+\rho } \right) , \quad x\ge 0 \end{aligned}$$

satisfies both (a2.) and (a3.).

### Example 2

Stochastic Ricker model (Högnäs 1997) is given by a density dependent branching process with the offspring distribution

$$\begin{aligned} p_\ell (x) = q_\ell e^{-\gamma x}, \end{aligned}$$

where $\gamma >0$ is a constant, $q_\ell$, $\ell \ge 1$ is a given probability distribution, and no offspring are produced with probability $1-e^{-\gamma x}$. This model satisfies the stochastic ordering assumption (a1.). The mean value of the distribution $q_\ell$ is denoted by $e^r$, to emphasize the relation to the deterministic Ricker model. With such notation,

$$\begin{aligned} m(x)=e^{r-\gamma x},\;\;f(x)=xe^{r-\gamma x}. \end{aligned}$$

Under normalization $m(0)=\rho$ and $m(1)=1$ this becomes

$$\begin{aligned} m(x)=\rho ^{1-x},\;\;f(x)=x \rho ^{1-x}. \end{aligned}$$

A direct calculation verifies the assumptions (a2.) and (a3.).

## Proof of Theorem 2

We will construct the process *Z* defined in (1) and the Galton–Watson process *Y* from (8) on a suitable probability space so that $Y_n\ge Z_n$ for all $n\in {\mathbb {N}}$ and the trajectories of these processes remain sufficiently close at least for relatively small *n*’s (Sect. 3.2). We will then show that *H* is twice continuously differentiable (Sect. 3.1) and use Taylor’s approximation to argue (Sect. 3.3) that

$$\begin{aligned} {{\overline{Z}}}_{n_1} - H({{\overline{Y}}}_{n_1}) = O_{\textsf{P}}(K^{-1/2} \log K), \quad \text {as }\, K\rightarrow \infty . \end{aligned}$$

This convergence combined with (11) implies the result. Below we will write *C* for a generic constant whose value may change from line to line.

### Properties of ${\textbf{H}}$

In this section we establish existence of the limit (9) under the standing assumptions and verify its smoothness. The proof of existence relies on a result on functional iteration, shown in Baker et al. (2020).

#### Lemma 3

(Baker et al. 2020, Lemma 1) Let $x_{m,n}$ be the sequence generated by the recursion

$$\begin{aligned} x_{m,n} = \rho x_{m-1,n}(1+C x_{m-1,n}), \quad m=1,\ldots ,n \end{aligned}$$

subject to the initial condition $x_{0,n}=x/\rho ^n>0$, where $\rho >1$ and $C\ge 0$ are constants. There exists a locally bounded function $\psi :{\mathbb {R}}_+\mapsto {\mathbb {R}}_+$ such that for any $n\in {\mathbb {N}}$

12 $$\begin{aligned} x_{m,n} \le \psi (x)\rho ^{m-n}, \quad m=1,\ldots ,n. \end{aligned}$$

Throughout we will use the notation $H_n(x):=f^n(x/\rho ^n)$.

#### Lemma 4

Under assumption (a3.) there exists a continuous function $H:{\mathbb {R}}_+\mapsto {\mathbb {R}}_+$ and a locally bounded function $g:{\mathbb {R}}_+\mapsto {\mathbb {R}}_+$ such that

$$\begin{aligned} \big |H_n(x) - H(x)\big |\le g(x) \rho ^{-n},\quad n\in {\mathbb {N}}. \end{aligned}$$

#### Proof

By assumption (a3.)

13 $$\begin{aligned} f(x) = \rho x + \int _0^x \int _0^t f''(s)ds dt \end{aligned}$$

and hence for any $x,y\in {\mathbb {R}}_+$

14 $$\begin{aligned} |f(y)-f(x)| \le \rho |y-x| +\frac{1}{2} \Vert f''\Vert _\infty |y^2-x^2| \le \rho \big (1 + C|y|\vee |x|\big )|y-x| \nonumber \\ \end{aligned}$$

with $C=\Vert f''\Vert _\infty /\rho$. Thus the sequence $x_{m,n}:= f^m(x/\rho ^n)$ satisfies

$$\begin{aligned} x_{m,n} = f (x_{m-1,n}) \le \rho \big (1 + C x_{m-1,n} \big ) x_{m-1,n} \end{aligned}$$

and $x_{0,n}=x/\rho ^n$. By Lemma 3 there exists a locally bounded function $\psi$ such that for any $n\in {\mathbb {N}}$

15 $$\begin{aligned} \big |f^m(x/\rho ^n)\big | \le \psi (x)\rho ^{m-n}, \quad m=1,\ldots ,n. \end{aligned}$$

The bound (14) also implies

16 $$\begin{aligned} \begin{aligned} \big |f^{m+1}(x/\rho ^{n+1}) - f^m (x/\rho ^n) \big |&= \big |f\circ f^{m}(x/\rho ^{n+1}) - f\circ f^{m-1} (x/\rho ^n) \big | \\&\quad \le \rho \big (1+ C F_{m,n}\big ) \big |f^m(x/\rho ^{n+1}) - f^{m-1} (x/\rho ^n) \big | \end{aligned}\nonumber \\ \end{aligned}$$

where, in view of (15),

$$\begin{aligned} F_{m,n}:= f^{m}(x/\rho ^{n+1})\vee f^{m-1} (x/\rho ^n) \le \psi (x) \rho ^{m-1-n}. \end{aligned}$$

Since *f* has bounded second derivative and $f'(0)=\rho$, cf. (13),

$$\begin{aligned} |f(x/\rho ^{n+1}) - x/\rho ^n| \le \frac{1}{2} \Vert f''\Vert _\infty (x/\rho )^2 \rho ^{-2n}. \end{aligned}$$

Plugging this bound into (16) and iterating *n* times we obtain

$$\begin{aligned}&\big |f^{n+1}(x/\rho ^{n+1}) - f^n (x/\rho ^n) \big | \le \big |f(x/\rho ^{n+1}) - x/\rho ^n\big |\, \rho ^n \prod _{m=1}^n \big (1+ C F_{m,n}\big ) \\&\quad \le \frac{1}{2} \Vert f''\Vert _\infty (x/\rho )^2 \rho ^{-2n}\rho ^n \prod _{m=1}^n \big (1+ C \psi (x) \rho ^{m-1-n}\big ) \le {\widetilde{g}}(x) \rho ^{-n} \end{aligned}$$

where we defined

$$\begin{aligned} {\widetilde{g}}(x):= \frac{1}{2} \Vert f''\Vert _\infty (x/\rho )^2 \prod _{k=1}^\infty \big (1+ C \psi (x) \rho ^{-k}\big ). \end{aligned}$$

Thus the limit $H(x)=\lim _{n\rightarrow \infty } f^n(x/\rho ^n)$ exists and satisfies the claimed bound with $g(x)={\widetilde{g}}(x)/(1-\rho ^{-1})$. Continuity of *H* follows since $H_n$ are continuous for each *n* and the convergence is uniform on compacts. $\square$

#### Corollary 5

*f* is topologically semiconjugate to its linearization at the origin:

$$\begin{aligned} H(x)=f\circ H(x/\rho ), \quad \forall x\in {\mathbb {R}}_+. \end{aligned}$$

#### Proof

Since *f* is continuous

$$\begin{aligned} H(x)=\lim _{n\rightarrow \infty }f^{n+1}(x/\rho ^{n+1}) = \lim _{n\rightarrow \infty } f\circ f^n((x/\rho )\rho ^{-n}) = f\circ H(x/\rho ). \end{aligned}$$

$$\square $$

The next lemma shows that *H* is strictly increasing in a vicinity of the origin and is therefore a local conjugacy.

#### Lemma 6

There exists an $a>0$ such that *H* is strictly increasing on [0, *a*] and

17 $$\begin{aligned} f(x) = H (\rho H^{-1}(x)), \quad x\in [0,H(a)]. \end{aligned}$$

#### Proof

Let $c:= \Vert f''\Vert _\infty$ and $r:= \rho /c$ then

$$\begin{aligned} f'(x) \ge \rho - c x>0, \quad \forall x\in [0,r). \end{aligned}$$

Since *f* is $\rho$-Lipschitz and $f(0)=0$, for any $j=1,\ldots ,n$ and $x\in [0,r)$,

$$\begin{aligned} f^{n-j}(x/\rho ^n)\le x/\rho ^j\in [0,r) \end{aligned}$$

and hence for all $x\in [0,r)$

$$\begin{aligned} H_n'(x)&= \prod _{j=1}^n \frac{1}{\rho }f'(f^{n-j}(x/\rho ^n)) \ge \prod _{j=1}^n \big (1-\frac{c}{\rho }f^{n-j}(x/\rho ^n) \big ) \\&\ge \prod _{j=1}^n \big (1-\frac{c}{\rho }x\rho ^{-j} \big ) \ge 1-\frac{c}{\rho }x \sum _{j=1}^n \rho ^{-j} \ge 1-\frac{c}{\rho -1} x, \end{aligned}$$

where we used the Bernoulli inequality. Thus we can choose a number $a\in (0,r)$ such that $H_n'(x)\ge 1/2$ for all $x\in [0,a]$. It then follows that for any $y>x$ in the interval [0, *a*]

$$\begin{aligned} H_n(y)-H_n(x) = \int _x^y H_n'(t)dt \ge \frac{1}{2} (y-x) >0. \end{aligned}$$

Taking the limit $n\rightarrow \infty$ implies that *H* is strictly increasing on [0, *a*]. Being continuous, *H* is invertible and (17) holds by Corollary 5. $\square$

#### Remark 4

Under additional assumption that *f* is strictly increasing on the whole ${\mathbb {R}}_+$, the function *H* is furthermore a global conjugacy, i.e. (17) holds on ${\mathbb {R}}_+$.

The next lemma establishes differentiability of *H*.

#### Lemma 7

*H* has continuous derivative

18 $$\begin{aligned} H'(x)= \prod _{j=1}^{\infty } \frac{1}{\rho }f'(H(x \rho ^{-j})),\quad \forall x\in {\mathbb {R}}_+ \end{aligned}$$

where the series converges uniformly on compacts.

#### Proof

Step 1. Let us first argue that the infinite product in (18)

19 $$\begin{aligned} G(x):= \prod _{j=1}^{\infty } \frac{1}{\rho }f'(H(x \rho ^{-j})) \end{aligned}$$

is well defined. By assumption (a3.), *f* is $\rho$-Lipschitz and hence $f^n$ is $\rho ^n$-Lipschitz. Consequently, $H_n$ is 1-Lipschitz for all $n\in {\mathbb {N}}$ and so is *H*. This will be used in the proof on several occasions. Let $c:=\Vert f''\Vert _\infty$ and $r:=\frac{1}{2}\rho /c$, then

20 $$\begin{aligned} f'(x) \ge \rho -cx>0, \quad \forall x\in [0,r]. \end{aligned}$$

For $x>0$ define the function $j(x):=[\log _\rho (x/r)]$. Then for any $j>j(x)$,

21 $$\begin{aligned} \begin{aligned} \Big |\log \frac{1}{\rho }f'(H(x \rho ^{-j}))\Big |&= -\log \frac{1}{\rho }f'(H(x \rho ^{-j})) \le -\log \Big (1-\frac{c}{\rho }H(x \rho ^{-j})\Big ) \\&{\mathop {\le }\limits ^{\dagger }}2 \frac{c}{\rho }H(x \rho ^{-j}) \le 2 \frac{c}{\rho }x \rho ^{-j} =: C x\rho ^{-j}, \end{aligned} \end{aligned}$$

where $\dagger$ holds since $-\log (1-u)\le 2u$ for all $u\in [0,\frac{1}{2}]$. The partial products in (19) can be written as

$$\begin{aligned} G_n(x)&:= \prod _{j=1}^{n} \frac{1}{\rho }f'(H(x \rho ^{-j})) \\&= \left( \prod _{j=1}^{j(x)} \frac{1}{\rho }f'(H(x \rho ^{-j})) \right) \exp \left( \sum _{j=j(x)+1}^n \log \frac{1}{\rho }f'(H(x \rho ^{-j}))\right) =: T(x)\exp (L_n(x)). \end{aligned}$$

In view of the estimate (21), $G_n(x)$ converges to $G(x):=T(x)\exp (L(x))$ for any $x\in {\mathbb {R}}_+$ where $L(x)=\lim _n L_n(x)$. Furthermore,

22 $$\begin{aligned} \begin{aligned} \big |G_n(x)-G(x)\big |&= |T(x)| \big |\exp (L_n(x))-\exp (L(x))\big | \\&\le \exp (L(x)\vee L_n(x))\big | L(x) -L_n(x) \big | \end{aligned} \end{aligned}$$

where we used the bound $|T(x)|\le 1$. For any $R>0$ and all $x\in [0,R]$ the estimate (21) implies

$$\begin{aligned} \big | L(x) -L_n(x) \big | = \sum _{j=n+1}^\infty \big |\log \frac{1}{\rho }f'(H(x \rho ^{-j}))\big | \le \sum _{j=n+1}^\infty C x\rho ^{-j} \le C R \frac{\rho ^{-n}}{\rho -1}, \end{aligned}$$

and thus, in view of the bound (22), we obtain

23 $$\begin{aligned} \sup _{x\le R} \big |G_n(x)-G(x)\big |\rightarrow 0. \end{aligned}$$

Since $G_n$ is continuous for any *n*, this uniform convergence implies that *G* is continuous as well.

Step 2. To show that *H*(*x*) is differentiable and to verify the claimed formula for the derivative, it remains to show that the sequence of derivatives

$$\begin{aligned} H_n'(x) = \prod _{j=1}^n \frac{1}{\rho }f'(f^{n-j}(x/\rho ^n)) \end{aligned}$$

converges to *G* uniformly on compacts. Fix an $R>0$, define $J(R)=[\log _\rho (R/r)]$ and, for $n>J(R)$, let

$$\begin{aligned} {\widetilde{P}}_n(x):= \prod _{j=1}^{J(R)} \frac{1}{\rho }f'(f^{n-j}(x/\rho ^n)),\quad P_n(x):= \prod _{j=J(R)+1}^n \frac{1}{\rho }f'(f^{n-j}(x/\rho ^n)) \end{aligned}$$

and

$$\begin{aligned} {\widetilde{Q}}_n(x):= \prod _{j=1}^{J(R)} \frac{1}{\rho }f'(H(x \rho ^{-j})), \quad Q_n(x):= \prod _{j=J(R)+1}^n \frac{1}{\rho }f'(H(x \rho ^{-j})). \end{aligned}$$

Since $\Vert f'\Vert _\infty =\rho$ all these functions are bounded by 1 and

$$\begin{aligned}&\big |H_n'(x) - G(x)\big |\le \big |H_n'(x) - G_n(x)\big | + \big |G_n(x) - G(x)\big | \\&\quad = \big |{\widetilde{P}}_n(x) P_n(x) - {\widetilde{Q}}_n(x) Q_n(x)\big | + \big |G_n(x) - G(x)\big | \\&\quad \le \big | P_n(x)- Q_n(x) \big | + \big |{\widetilde{P}}_n(x) - {\widetilde{Q}}_n(x)\big | + \big |G_n(x) - G(x)\big |. \end{aligned}$$

Since $f'$ is continuous and the convergence $H_n\rightarrow H$ is uniform on compacts, it follows that

$$\begin{aligned} \sup _{x\le R}\big |{\widetilde{P}}_n(x) - {\widetilde{Q}}_n(x)\big | = \sup _{x\le R}\left| \prod _{j=1}^{J(R)} \frac{1}{\rho }f'(H_{n-j}(x\rho ^{-j})) - \prod _{j=1}^{J(R)} \frac{1}{\rho }f'(H(x \rho ^{-j})) \right| \xrightarrow [n\rightarrow \infty ]{}0, \end{aligned}$$

and hence, to complete the proof, we need to show that

24 $$\begin{aligned} \sup _{x\le R}\big | P_n(x) - Q_n(x)\big |\xrightarrow [n\rightarrow \infty ]{}0. \end{aligned}$$

To this end, in view of Corollary 5,

$$\begin{aligned} H(x\rho ^{-j})&= f\circ H(x\rho ^{-j-1})) = f^2\circ H(x\rho ^{-j-2})) = \ldots \\&=f^{n-j}\circ H(x\rho ^{-j-(n-j)})= f^{n-j}\circ H(x\rho ^{ - n }) \end{aligned}$$

and hence

$$\begin{aligned} P_n(x) - Q_n(x) = \prod _{j=J(R)+1}^n \frac{1}{\rho }f'(f^{n-j}(x \rho ^{-n})) - \prod _{j=J(R)+1}^n \frac{1}{\rho }f'\big (f^{n-j}\big (H(x \rho ^{-n})\big )\big ). \end{aligned}$$

Consequently, for all $x\in (0,R]$,

25 $$\begin{aligned} \begin{aligned}&\big |\log P_n(x) - \log Q_n(x)\big | \\&\quad \le \sum _{j=J(R)+1}^n \Big | \log \frac{1}{\rho }f'(f^{n-j}(x\rho ^{-n})) - \log \frac{1}{\rho }f'\big (f^{n-j}\big (H(x\rho ^{ - n })\big )\big )\Big | \\&\quad {\mathop {\le }\limits ^{\dagger }}\frac{1}{\rho - cr} \Vert f''\Vert _\infty \sum _{j=J(R)+1}^n \big | f^{n-j}(x\rho ^{-n}) - f^{n-j}\big (H(x\rho ^{ - n })\big ) \big | \\&\quad \le \frac{1}{\rho - cr} \Vert f''\Vert _\infty \sum _{j=1}^n \rho ^{ n-j}\big | x\rho ^{-n} - H(x\rho ^{ - n }) \big | \le C \rho ^n \big | \rho ^{-n} x - H(x\rho ^{ - n }) \big | \\&\quad = C \rho ^n \big | H\circ H^{-1}( x\rho ^{-n} ) - H(x\rho ^{ - n }) \big | {\mathop {\le }\limits ^{\ddagger }}C \big | \rho ^n H^{-1}( x\rho ^{-n} ) - x \big |. \end{aligned} \end{aligned}$$

Here the bound $\dagger$ holds since for $j> J(R)$ both arguments of $f'$ are smaller than *r* and thus (20) applies. The inequality $\ddagger$ is true since *H* is 1-Lipschitz. The inverses in the last line of (25) are well defined for $n\ge k:=[\log _\rho (R/H(a))]+1$ where *a* is the constant guaranteed by Lemma 6. Moreover, for all such *n*

26 $$\begin{aligned}&\big | \rho ^n H^{-1}( x\rho ^{-n}) - x \big | = \rho ^k \big | \rho ^{n-k} H^{-1}( x\rho ^{-k}\rho ^{-(n-k)}) - x\rho ^{-k} \big | \nonumber \\&\quad = \rho ^k \big | H^{-1}\circ f^{n-k}( x\rho ^{-k}\rho ^{-(n-k)} ) - x \rho ^{-k} \big | = \rho ^k \big | H^{-1}\circ H_{n-k}(x\rho ^{-k}) - x \rho ^{-k} \big | \xrightarrow [n\rightarrow \infty ]{}0. \end{aligned}$$

Moreover, the sequence of functions $D_n(x):= \rho ^n H^{-1}( x\rho ^{-n})$ is decreasing on [0, *R*] for all *n* large enough:

$$\begin{aligned} D_{n+1}(x) = \rho ^n \rho H^{-1}( x\rho ^{-n-1}) = \rho ^n H^{-1}\circ f ( x\rho ^{-n-1})\le \rho ^n H^{-1} ( x\rho ^{-n }) = D_n(x), \end{aligned}$$

where the inequality holds since $H^{-1}$ is increasing near the origin. It follows now from Dini’s theorem that the convergence in (26) is uniform:

$$\begin{aligned} \sup _{x\le R} \big | \rho ^n H^{-1}( x\rho ^{-n} ) - x \big |\xrightarrow [n\rightarrow \infty ]{}0. \end{aligned}$$

The convergence in (24) holds since both $Q_n$ and $P_n$ are bounded by 1 and

$$\begin{aligned} \sup _{x\le R}\big | P_n(x) - Q_n(x)\big | \le \sup _{x\le R}\big |P_n(x) \vee Q_n(x)\big | \sup _{x\le R}\big | \log P_n(x) - \log Q_n(x)\big | \xrightarrow [n\rightarrow \infty ]{}0. \end{aligned}$$

$$\square $$

#### Lemma 8

*H* has continuous second derivative

27 $$\begin{aligned} H''(x) = H'(x) \sum _{i=1}^\infty \frac{f''(H(x \rho ^{-i}))}{ f'(H(x \rho ^{-i}))} H'(x \rho ^{-i})\rho ^{-i}. \end{aligned}$$

#### Proof

The partial products in (18)

$$\begin{aligned} G_n(x): =\prod _{j=1}^n \frac{1}{\rho }f'(H(x \rho ^{-j})) \end{aligned}$$

satisfy

$$\begin{aligned} G'_n(x)&= \sum _{i=1}^n\left( \prod _{j=1, j\ne i}^n \frac{1}{\rho }f'(H(x \rho ^{-j}))\right) \frac{1}{\rho }f''(H(x \rho ^{-i}))H'(x \rho ^{-i}) \rho ^{-i} \\&= G_n(x) \sum _{i=1}^n \frac{f''(H(x \rho ^{-i}))}{ f'(H(x \rho ^{-i}))} H'(x \rho ^{-i}) \rho ^{-i}, \end{aligned}$$

where the convention $0/0=0$ is used. By assumption (a3.), $f''/f'$ is bounded uniformly on a vicinity of the origin. $H'$ is continuous by Lemma 7 and therefore is bounded on compacts. Hence the series is compactly convergent. By Lemma 7, so is $G_n$. Thus $G'_n(x)$ converges compactly, its limit is continuous and coincides with $H''(x)$. $\square$

### The auxiliary Galton–Watson process

Let $(U_{n,j}: n\in {\mathbb {N}}, j\in {\mathbb {Z}}_+)$ be an array of i.i.d. *U*([0, 1]) random variables and define

$$\begin{aligned} \xi _{n,j}(x) = F^{-1}_x(U_{n,j}):= \min \big \{t\ge 0: F_x(t)\ge U_{n,j}\big \}, \end{aligned}$$

where $F_x(t)$ is the offspring distribution function when the population density is *x*, cf. assumption (a1.). Then ${\textsf{P}}(\xi _{n,j}(x)=k)=p_k(x)$ for all $k\in {\mathbb {Z}}_+$. Let $\eta _{n,j}:=\xi _{n,j}(0)$. By assumption (a1.)

28 $$\begin{aligned} \xi _{n,j}(x) \le \eta _{n,j}\quad \forall x\in {\mathbb {R}}_+,\ n,j\in {\mathbb {N}}. \end{aligned}$$

Let $Z=(Z_n, n\in {\mathbb {Z}}_+)$ and $Y=(Y_n, n\in {\mathbb {Z}}_+)$ be processes generated by the recursions

$$\begin{aligned} Z_n = \sum _{j=1}^{Z_{n-1}}\xi _{n,j}({{\overline{Z}}}_{n-1})\quad \text {and}\quad Y_n = \sum _{j=1}^{Y_{n-1}} \eta _{n,j} \end{aligned}$$

started from the same initial conditions $Z_0=Y_0=1$. By construction these processes coincide in distribution with (1) and (8) respectively. Moreover, in view of (28), by induction

29 $$\begin{aligned} Z_n \le Y_n, \quad \forall n\in {\mathbb {Z}}_+. \end{aligned}$$

### The approximation

In view of (11),

$$\begin{aligned} {{\overline{Y}}}_{n_1} - W\rho ^{-\{\log _\rho K\}} = \rho ^{-\{\log _\rho K\}}\big (\rho ^{-n_1} Y_{n_1}- W\big ) =O_{\textsf{P}}(\rho ^{-n_1/2}) = O_{\textsf{P}}(K^{-1/2}). \end{aligned}$$

Since *H* has continuous derivative it follows that

$$\begin{aligned} H( {{\overline{Y}}}_{n_1})- H(W\rho ^{-\{\log _\rho K\}}) = O_{\textsf{P}}(K^{-1/2}). \end{aligned}$$

Thus to prove the assertion of Theorem 2 it remains to show that

$$\begin{aligned} {{\overline{Z}}}_{n_1} - H({{\overline{Y}}}_{n_1}) = O_{\textsf{P}}(K^{-1/2}\log K), \quad K\rightarrow \infty . \end{aligned}$$

The process ${{\overline{Y}}}_n = K^{-1} Y_n$ satisfies

$$\begin{aligned} {{\overline{Y}}}_n = \rho {{\overline{Y}}}_{n-1} + \frac{1}{K} \sum _{j=1}^{Y_{n-1}}(\eta _{n,j}-\rho ). \end{aligned}$$

By Taylor’s approximation and in view of Corollary 5

30 $$\begin{aligned} \begin{aligned} H({{\overline{Y}}}_n)&= H(\rho {{\overline{Y}}}_{n-1}) + H'(\rho {{\overline{Y}}}_{n-1})\frac{1}{K} \sum _{j=1}^{Y_{n-1}}(\eta _{n,j}-\rho ) + R_n(K) \\&= f(H( {{\overline{Y}}}_{n-1})) + H'(\rho {{\overline{Y}}}_{n-1})\frac{1}{K} \sum _{j=1}^{Y_{n-1}}(\eta _{n,j}-\rho ) + R_n(K) \end{aligned} \end{aligned}$$

where

31 $$\begin{aligned} R_n(K):= \frac{1}{2} H''(\theta _{n-1}(K))\left( \frac{1}{K} \sum _{j=1}^{Y_{n-1}}(\eta _{n,j}-\rho )\right) ^2 \end{aligned}$$

with $$\theta _{n-1}(K)\ge 0$$ satisfying

32 $$\begin{aligned} \big |\theta _{n-1}(K) - \rho {{\overline{Y}}}_{n-1}\big |\le \left| \frac{1}{K} \sum _{j=1}^{Y_{n-1}}(\eta _{n,j}-\rho )\right| . \end{aligned}$$

Since $\Vert f'\Vert _\infty = \rho$ is assumed, *f* is $\rho$-Lipschitz. By subtracting equation (5) from (30) we obtain the bound for $\delta _n:= | H({{\overline{Y}}}_n)-{{\overline{Z}}}_n|$:

33 $$\begin{aligned} \delta _n \le \rho \delta _{n-1} + \big |\varepsilon ^{(1)}_n\big | + \big |\varepsilon ^{(2)}_n\big | +\big |\varepsilon ^{(3)}_n\big | + |R_n(K)| \end{aligned}$$

subject to $\delta _0 = |H(1/K)-1/K|$, where we defined

$$\begin{aligned} \varepsilon ^{(1)}_n&= \Big (H'(\rho {{\overline{Y}}}_{n-1})-1\Big )\frac{1}{K} \sum _{j=1}^{Y_{n-1}}(\eta _{n,j}-\rho ), \\ \varepsilon ^{(2)}_n&= \frac{1}{K} \sum _{j=1}^{Z_{n-1}}\Big ((\eta _{n,j}-\rho ) - (\xi _{n,j}({{\overline{Z}}}_{n-1})-m({{\overline{Z}}}_{n-1}))\Big ),\\ \varepsilon ^{(3)}_n&= \frac{1}{K} \sum _{j=Z_{n-1}+1}^{Y_{n-1}}(\eta _{n,j}-\rho ). \end{aligned}$$

Consequently,

$$\begin{aligned} \delta _n \le \rho ^n \delta _0 + \sum _{j=1}^n \rho ^{n-j} \Big (\big |\varepsilon ^{(1)}_j\big | + \big |\varepsilon ^{(2)}_j\big | +\big |\varepsilon ^{(3)}_j\big | + |R_j(K)| \Big ) \end{aligned}$$

and it is left to show that the contribution of each term at time $n_1=[\log _ \rho K]$ is of order $O_{\textsf{P}}(K^{-1/2}\log K)$ as $K\rightarrow \infty$.

#### Contribution of the initial condition

Since $H(0)=0$ and, by (18), $H'(0)=1$, Taylor’s approximation implies that for all *K* large enough

$$\begin{aligned} \delta _0 = |H(1/K)-1/K| \le \frac{1}{2}\sup _{x\le 1}|H''(x)| K^{-2} = CK^{-2} \end{aligned}$$

and, consequently, $$ |\rho ^{n_1} \delta _0| \le CK^{-1}. $$

#### Contribution of $R_n(K)$

To estimate the residual, defined in (31), let us show first that the family of random variables

34 $$\begin{aligned} \max _{m\le n_1} \Big |H''(\theta _m(K))\Big | \end{aligned}$$

is bounded in probability as $K\rightarrow \infty$. By equation (32),

$$\begin{aligned} {\textsf{E}}\theta _{n-1}(K)&\le \ \rho {\textsf{E}}{{\overline{Y}}}_{n-1} + {\textsf{E}}\left| \frac{1}{K} \sum _{j=1}^{Y_{n-1}} \big (\eta _{n,j}-\rho \big )\right| \\&\le \rho {\textsf{E}}{{\overline{Y}}}_{n-1} + \frac{1}{K} \sqrt{{\textsf{E}}Y_{n-1} \sigma ^2(0)}\le \frac{1}{K}\rho ^n + \frac{1}{K} \sqrt{ \rho ^{ n} \sigma ^2(0)} \\&\le K^{-1} \rho ^n + C K^{-1}\rho ^{n/2} \le 2CK^{-1} \rho ^n. \end{aligned}$$

If $H''$ is bounded then (34) is obviously bounded. Let us proceed assuming that $H''$ is unbounded. Define $\psi (M):= \max _{x\le M} |H''(x)|.$ By continuity, $\psi (M)$ is finite, continuous and increases to $\infty$. Let $\psi ^{-1}$ be its generalized inverse

$$\begin{aligned} \psi ^{-1}(t)=\inf \{x\ge 0: \psi (x)\ge t\}. \end{aligned}$$

Since $\psi$ is continuous and unbounded, $\psi ^{-1}$ is nondecreasing (not necessarily continuous) and $\psi ^{-1}(t)\rightarrow \infty$ as $t\rightarrow \infty$. Then for any $R\ge 0$, by the union bound,

$$\begin{aligned}&{\textsf{P}}\Big (\max _{m\le n_1} |H''(\theta _m(K)|\ge R\Big )\le {\textsf{P}}\Big (\max _{m\le n_1} \psi (\theta _m(K))\ge R\Big ) \\&\quad \le \sum _{m=1}^{n_1} {\textsf{P}}\Big ( \psi (\theta _m(K))\ge R\Big ) \le \sum _{m=1}^{n_1} {\textsf{P}}\Big ( \theta _m(K) \ge \psi ^{-1}(R)\Big ) \\&\quad \le \sum _{m=1}^{n_1} \frac{{\textsf{E}}\theta _m(K)}{ \psi ^{-1}(R)} \le \frac{1}{ \psi ^{-1}(R)} \sum _{m=1}^{n_1} 2C K^{-1} \rho ^m \le \frac{\rho }{\rho -1}\frac{2C}{ \psi ^{-1}(R)}\xrightarrow [R\rightarrow \infty ]{}0. \end{aligned}$$

This proves that (34) is bounded in probability. The contribution of $R_n(K)$ in (33) can now be bounded as

$$\begin{aligned} \left| \sum _{m=1}^{n_1} \rho ^{n_1-m} R_m(K)\right| \le \max _{j\le n_1}\Big | H''(\theta _j(K))\Big | \sum _{m=1}^{n_1} \rho ^{n_1-m} \left( \frac{1}{K} \sum _{j=1}^{Y_{m-1}}(\eta _{m,j}-\rho )\right) ^2 \end{aligned}$$

where

$$\begin{aligned}&{\textsf{E}}\sum _{m=1}^{n_1} \rho ^{n_1-m} \left( \frac{1}{K} \sum _{j=1}^{Y_{m-1}}(\eta _{m,j}-\rho )\right) ^2 \\&\quad = \sum _{m=1}^{n_1} \rho ^{n_1-m} \frac{1}{K^2} {\textsf{E}}Y_{m-1} \sigma ^2(0) \le \sum _{m=1}^{n_1} \rho ^{n_1-m} \frac{1}{K^2} \rho ^{m} \sigma ^2(0) \le C K^{-1}\log K. \end{aligned}$$

Hence

$$\begin{aligned} \left| \sum _{m=1}^{n_1} \rho ^{n_1-m} R_m(K)\right| = O_{\textsf{P}}(1) O_{\textsf{P}}(K^{-1} \log K) = O_{\textsf{P}}(K^{-1} \log K). \end{aligned}$$

#### Contribution of $\varepsilon ^{(3)}$

By conditional independence of $\eta _{n,j}$’s

$$\begin{aligned} {\textsf{E}}\big (\varepsilon ^{(3)}_m\big )^2 = \frac{\sigma ^2(0)}{K } {\textsf{E}}({{\overline{Y}}}_{m-1}-{{\overline{Z}}}_{m-1}). \end{aligned}$$

In view of (29), the sequence $D_m:= {{\overline{Y}}}_{m}-{\overline{Z}}_{m}\ge 0$ satisfies

$$\begin{aligned} {\textsf{E}}D_m&= \frac{1}{K}{\textsf{E}}\left( \sum _{j=1}^{Y_{m-1}}\eta _{m,j}-\sum _{j=1}^{Z_{m-1}}\xi _{m,j}({{\overline{Z}}}_{m-1})\right) \\&= \frac{1}{K}{\textsf{E}}\sum _{j=Z_{m-1}+1}^{Y_{m-1}}\eta _{m,j}+\frac{1}{K}{\textsf{E}}\sum _{j=1}^{Z_{m-1}}\big (\eta _{m,j}-\xi _{m,j}({{\overline{Z}}}_{m-1})\big ) \\&= \rho {\textsf{E}}D_{m-1} + \frac{1}{K}{\textsf{E}}\sum _{j=1}^{Z_{m-1}} \big (\rho -m({{\overline{Z}}}_{m-1})\big ) \\&= \rho {\textsf{E}}D_{m-1} + {\textsf{E}}{{\overline{Z}}}_{m-1} \big (\rho -m({{\overline{Z}}}_{m-1}) \big ) \\&= \rho {\textsf{E}}D_{m-1} + {\textsf{E}}\big (\rho {{\overline{Z}}}_{m-1}-f({{\overline{Z}}}_{m-1})\big ) \\&\le \rho {\textsf{E}}D_{m-1} + \frac{1}{2} \Vert f''\Vert _\infty {\textsf{E}}{{\overline{Z}}}_{m-1}^2 \\&\le \rho {\textsf{E}}D_{m-1} + C K^{-2} \rho ^{2m}, \end{aligned}$$

where the last bound holds in view of (29) and the well known formula for the second moment of the Galton–Watson process. Since $D_0=0$ it follows that

$$\begin{aligned} {\textsf{E}}D_m \le \sum _{\ell =1}^m \rho ^{m-\ell } C K^{-2} \rho ^{2\ell } \le C K^{-2}\rho ^{2m}. \end{aligned}$$

Hence the contribution of $\varepsilon ^{(3)}$ in (33) is bounded by

$$\begin{aligned} {\textsf{E}}\Big |\sum _{m=1}^{n_1} \rho ^{n_1-m} \varepsilon ^{(3)}_m\Big |&\le C \sum _{m=1}^{n_1} \rho ^{n_1-m} K^{-1/2} \sqrt{{\textsf{E}}D_m} \\&\le C \sum _{m=1}^{n_1} \rho ^{n_1-m} K^{-1/2} K^{-1}\rho ^{ m} \le C K^{-1/2} \log K. \end{aligned}$$

#### Contribution of $\varepsilon ^{(2)}$

By assumption (a2.),

$$\begin{aligned} {\textsf{E}}\big (\varepsilon ^{(2)}_m\big )^2&= K^{-2}{\textsf{E}}\sum _{j=1}^{Z_{m-1}} \big ( \eta _{m,j} -\xi _{m,j}({{\overline{Z}}}_{m-1}) - \big (\rho -m({{\overline{Z}}}_{m-1})\big ) \big )^2 \\&\le K^{-2}{\textsf{E}}\sum _{j=1}^{Z_{m-1}} \big (\eta _{m,j} -\xi _{m,j}({{\overline{Z}}}_{m-1})\big )^2 {\mathop {\le }\limits ^{\dagger }} K^{-2}{\textsf{E}}\sum _{j=1}^{Z_{m-1}} (m_2(0) - m_2({{\overline{Z}}}_{m-1})\big ) \\&\le K^{-2}{\textsf{E}}\sum _{j=1}^{Z_{m-1}} L {{\overline{Z}}}_{m-1} \le C K^{-3}\rho ^{2m} \end{aligned}$$

where $\dagger$ holds by (28). Hence $\varepsilon ^{(2)}$ contributes

$$\begin{aligned} {\textsf{E}}\Big |\sum _{m=1}^{n_1} \rho ^{n_1-m} \varepsilon ^{(2)}_m\Big | \le C \sum _{m=1}^{n_1} \rho ^{n_1-m} K^{-3/2}\rho ^{ m} \le C K^{-1/2}\log K. \end{aligned}$$

#### Contribution of $\varepsilon ^{(1)}$

The function $g(x): = H'(x)-1$ is continuously differentiable with $g(0)=0$ and thus Taylor’s approximation gives

$$\begin{aligned} \varepsilon ^{(1)}_n =\, g(\rho {{\overline{Y}}}_{n-1})\frac{1}{K} \sum _{j=1}^{Y_{n-1}}(\eta _{n,j}-\rho ) = g'(\zeta _{n-1}(K)) \rho {{\overline{Y}}}_{n-1}\frac{1}{K} \sum _{j=1}^{Y_{n-1}}(\eta _{n,j}-\rho ) \end{aligned}$$

where $\zeta _{n-1}(K)$ satisfies $0\le \zeta _{n-1}(K)\le \rho {{\overline{Y}}}_{n-1}$. Here

$$\begin{aligned}&{\textsf{E}}\Big |{{\overline{Y}}}_{n-1}\frac{1}{K} \sum _{j=1}^{Y_{n-1}}(\eta _{n,j}-\rho )\Big | \\&\quad \le \left( {\textsf{E}}\big ({{\overline{Y}}}_{n-1}\big )^2\right) ^{1/2} \left( {\textsf{E}}\Big (\frac{1}{K} \sum _{j=1}^{Y_{n-1}}(\eta _{n,j}-\rho )\Big )^2 \right) ^{1/2} \\&\quad \le \big (K^{-2}\rho ^{2n})^{1/2} \Big ( K^{-2} {\textsf{E}}Y_{n-1} \sigma ^2(0) \Big )^{1/2} \le C K^{-2} \rho ^{3/2n}. \end{aligned}$$

It follows that

$$\begin{aligned} {\textsf{E}}\sum _{m=1}^{n_1} \rho ^{n_1-m} \Big |{{\overline{Y}}}_{m-1}\frac{1}{K} \sum _{j=1}^{Y_{m-1}}(\eta _{m,j}-\rho )\Big | \le \sum _{m=1}^{n_1} \rho ^{n_1-m} C K^{-2} \rho ^{3/2m} \le C K^{-1/2}. \end{aligned}$$

It is then argued as in Sect. 3.3.2 that

$$\begin{aligned} \sum _{m=1}^{n_1} \rho ^{n_1-m} \varepsilon ^{(1)}_m = O_{\textsf{P}}(1) O_{\textsf{P}}(K^{-1/2}) =O_{\textsf{P}}(K^{-1/2}). \end{aligned}$$

## Footnotes

1 The usual notations for probabilistic orders is used throughout. In particular, for a sequence of random variables $\zeta (K)$ and a sequence of numbers $\alpha (K)\searrow 0$ as $K\rightarrow \infty$, the notation $\zeta (K)=O_{\textsf{P}}(\alpha (K))$ means that the sequence $\alpha (K)^{-1}\zeta (K)$ is bounded in probability.

2 [*x*] and $\{x\}=x-[x]$ denote the integer and fractional part of $x\in {\mathbb {R}}_+$.

3 $$\Vert f\Vert _\infty =\sup _x |f(x)|$$
